# A Hybrid Framework for Direct CO_2_ Emissions Quantification in China’s Construction Sector

**DOI:** 10.3390/ijerph182211965

**Published:** 2021-11-15

**Authors:** Adedayo Johnson Ogungbile, Geoffrey Qiping Shen, Ibrahim Yahaya Wuni, Jin Xue, Jingke Hong

**Affiliations:** 1 Department of Building and Real Estate, The Hong Kong Polytechnic University, Hong Kong, China; geofreyq.shen@polyu.edu.hk (G.Q.S.); ibrahim.wuni@connect.polyu.hk (I.Y.W.); jin.xue@connect.polyu.hk (J.X.); 2 School of Construction Management and Real Estate, Chongqing University, Chongqing 400045, China; hongjingke@cqu.edu.cn

**Keywords:** direct CO_2_ emissions, fossil fuel, energy consumption, regional construction industry, econometric analysis

## Abstract

Carbon emission quantifications in China are not consistent, with many standards and methods having been used over the years. This study identified the non-consideration of China-specific technology and databases as a factor limiting comprehensive quantification. The study aimed to comprehensively quantify regional direct CO_2_ emission in the industry using a hybrid of economic and environmental data. We retrieved nineteen (19) sets of fossil fuel and electricity data from provincial energy yearbooks between 1997 and 2015 for the study. To generate regression models for each of the six regional construction industries in China, the study further integrated the results with three sets of econometric data: total annual construction output, cement, and steel product yearly consumption data. The study identified the North China region as the main source of direct CO_2_ emission with over 30%, while Southeast China contributed the least. While there is a gradual shift to other energy sources, the study identified coal and crude oil to remain as the main energy sources in the industry. Cement and steel data exhibited a significant predictive relationship with CO_2_ emissions in five regional construction industries. The study identified the need to have policies tailored to technological improvements to enhance renewable energy generation and usage in the industry. The models developed in this study could be used to generate initial quantifications of carbon emissions in construction industries with similar carbon-emitting characteristics for carbon tracking, and energy policies for decision making. However, the three economic indicators used in the study could be extended to generate more robust models in future research.

## 1. Introduction

Recently, issues of climate change have dominated global discussions. In 2016, the United Nations set climate change as the thirteenth of the seventeen Sustainable Development Goals (SDG) [1]. The American Chemical Society (ACS) in 2017 estimated carbon dioxide (CO_2_) production in the present century to be 408.8 parts per million (ppm), 46% greater than the average concentration in the last two centuries, with a value of 280 ppm [2]. The global upward trend in CO_2_ production, coupled with visible biological degradations, could be related to climate change [3]. Over the years, the Intergovernmental Panel on Climate Change (IPCC) has reported the alarming dangers that loom for humankind and the environment at large because of anthropogenic climate change [4]. Many of the risks forecasted have occurred and intensified due to weather immoderations, such as extreme drought, hurricanes, flooding, and increased global heat [5]. The American Meteorological Society [6] described CO_2_ emissions as having the most significant contribution to global climate change, with China being the most critical contributor to global CO_2_ emissions.

The National Development and Reform Commission (NDRC) report in 2014 estimated CO_2_ emissions from China at 10,300 MtCO_2_ in 2013 [7]. The report further predicted CO_2_ emissions would increase in the following decades. Hong et al. [8] attributed China’s rise in CO_2_ emissions to the challenge of energy consumption and substantial environmental burdens due to rapid urbanization. In 2014, China’s urbanization rate reached 54.77% and was expected to increase to about 60% in 2020 [1]. The increasing urbanization growth, coupled with the construction industry’s expanding scope in providing amenities and facilities, presents considerable energy demands and energy-related CO_2_ emission challenges [9,10].

The past literature focused on quantifying the construction industry’s CO_2_ emissions from the sector’s upstream economic structure and operational phase [11,12]. Many studies argued the non-significance of estimating onsite-generated direct CO_2_ emissions in the industry, as most CO_2_ emissions in the sector are generated during operational phase usage [13,14]. Some studies argued the insignificance of direct CO_2_ emissions, as low as 2% of all generated CO_2_ emissions in the sector [13]. However, Sartori and Hestnes [15] estimated that direct CO_2_ emissions could be as high as 38% and 46% in conventional and low-energy buildings, respectively. With the trend of international focus on CO_2_ emissions, it is essential to estimate the direct CO2 emissions from one of the largest global construction sectors to determine its effects on the overall CO_2_ emissions profile.

In addition, the selection of datasets in computing the Chinese construction sector emission quantities has thwarted sound policy decisions to ensure environmental sustainability [16]. Many studies have argued the unreliability of CO_2_ emissions figures in the Chinese construction sector due to the lack of local technological considerations and double counting effects [17,18]. Therefore, it is essential to investigate CO_2_ emissions originating from the Chinese construction sectors’ direct injections, from onsite construction activities to downstream sources of the sector’s supply chain. Therefore, the paper aims to holistically quantify direct CO_2_ emissions from China’s construction sector activities at national and sub aggregated regional levels (Table 1). The study adopted a mixed approach to quantifying direct CO_2_ emissions during the sector’s construction stage. Unlike many studies, this paper uses regression analysis to combine parametric data with the CO_2_ emissions in the Chinese construction sector from a careful choice of databases. Models generated from the regression analysis are targeted at giving preliminary estimates of direct CO_2_ emissions in construction sectors if the variables used in the study are available. Policymakers and stakeholders could use the preliminary results for a fast decision-making process while calculating detailed estimates. Additionally, construction firms and managers could use the initial assessment generated to optimize fuel usage and process improvements on construction sites.

The article is organized into six sections, including the introduction. The second section summarizes the extant literature. The third section presents the methodology used for the regional CO_2_ emissions analysis and subsequent regression analysis. Next, the results of the study are discussed in the fourth section. The fifth section flows logically from the previous by presenting the research policy implications of the results discussed in the fourth section. Lastly, the sixth section gives the concluding parts of the article.

## 2. Literature Review

China has become a force to reckon with in global discussions and has grown its economy from just being the most populated nation to one with the world’s most promising technological hubs and one of the world’s fastest-growing economies [19]. China’s growth over the years is evident in its global contributions and the per capita GDP growth of the country. China experienced an increased per capita GDP from USD 939 in 2000 to USD 10,004 in 2019, with a projected growth of 10.95% in 2021 [20]. With the domestic growth in financial capacities, global relevance, and recognition as a world power, there has been a corresponding record development in many sectors of China’s economic structures. China’s average 2020 monthly industrial growth rate was 7.3%, compared to negative −4.8% and −4.2% in the United Kingdom and the USA, respectively [21]. The continued growth in China’s industrial, construction, and manufacturing processes has raised questions about sustainability and climate change.

The advocacy for a need to re-strategize for a sustainable environment championed by the IPCC led many countries to adopt new measures against the indiscriminate discharge of GHG emissions. Since the declaration of the international state of emergency on climate change and sustainable advocacy, efforts have been made in many developed countries. Countries such as the United States, the United Kingdom, Japan, and China have developed many policies to reduce GHG emission stocks in their region. According to Chen et al. [22], emission intensities are lower in developed countries than in developing countries. Since the dawn of the industrial revolution, China, coupled with the joining of the WTO, has continued to increase its industrial production and construction activities, making it the world’s largest developing nation [23].

Since the ratification in Kyoto, Japan, in 1997, 192 countries have adopted the convention’s recommendations regarding global warming. China is one such country that took the advice of the Kyoto protocol [24,25]. China was among the 36 countries that fully participated in the protocol’s first round of commitments between 2008 and 2012 by reducing GHG production. One of the Chinese government’s critical efforts was establishing the National Development and Reforms Commission (NDRC) in 2004 [26]. According to Chen et al. [26], “the NDRC’s functions are to study and formulate policies for economic and social development, maintain the balance of economic development, and to guide the restructuring of the economic system of Mainland China.” In China, the NDRC is a champion of industrial reform policies. Some of the critical reforms include raising energy-saving requirements for industries and corporations, with relevant authorities in the amendment of the 2011 edition of the Guideline Catalogue for Industrial Restructuring. The guideline underlines the importance of upgrading the industrial sector, increasing energy-saving requirements, and ultimately reducing GHG emissions.

The NDRC, in its 12th five-year plan period, issued a national Gas Development Plan targeted at optimizing energy structures in China through the promotion of the use of clean fossil fuels. The policy resulted in the number of towns and city residents’ using natural gas to rise by 18% in 2012. The agency announced plans to increase China’s development of non-fossil-fuel consumption. China increased its investment in clean and renewable energy sources, with almost 62 billion RMB invested in wind power, 78 billion RMB in nuclear power, and 127.7 billion RMB in hydropower stations [27]. Studies have shown that China’s dependence on coal as an energy source (a reflectance from the construction industry) is gradually reducing, with more investments made towards renewable energy sources and achieving carbon neutrality by 2060. The commitment to achieving non-fossil fuel energy consumption of 20% by 2030 reflects China’s stance on climate change [28].

In 2009, at the Copenhagen conference, China, in conjunction with other member countries of the United Nations Framework Convention on Climate Change (UNFCCC), agreed to reduce carbon emissions per unit of GDP by 40–45% of the 2005 levels by 2020. The Copenhagen accord endorsed the Kyoto protocol and was drafted in parts. The USA wrote one part of the agreement while the BASIC (China, Brazil, India, and South Africa) countries jointly wrote the other piece. The accord recognized climate change as a significant challenge to humans. It emphasized: “strong political will to urgently combat climate change following the principle of common but differentiated responsibilities and respective capabilities.” Although a few countries called out the legality of the accord, China, throughout the years, remained resolute in fulfilling its part in reducing carbon intensity by 40–45% of the 2005 level [27]. Furthermore, China has initiated a low-carbon city pilot project to reduce the carbon emissions in pilot cities [29] and has promised to reach the peak of carbon emissions no later than 2030 [22,30]. Therefore, the Chinese government regards climate change as a fundamental issue and has proposed a series of binding emission reduction targets [31].

However, with a promise of increasing the non-fossil-fuel share in primary energy consumption by 15% in 2020 through the Development Strategy Action Plan (2014–2020), China is still a distance away from expectations in combatting climate change. The United Nations Environmental Program (UNEP), in its preliminary investigations in 2010, reported an emission gap between the emissions necessary to obtain a “likely” global GHG emissions reduction (66% probability) and the emissions reduction promised at the accord signing. This discrepancy is vast in China, with the overall highest emission discharge. In a report by the International Energy Agency (IEA) in 2015, China was found to have the largest share of GHG emissions from domestic and international trade [32]. The construction industry has a significant share, being the largest exporting sector in China. Simulated predictions from the IEA database indicated that China’s GHG emissions would grow by more than twice the world’s average [7].

Rising to the occasion, the Chinese government has made numerous policies to cushion the effects of climate change and reduce China’s generated emissions in the global stock. These policies cut across many industrial formations and energy uses. However, this study observes that China’s construction industry lacks specific vital policies to improve the industry’s CO_2_ emissions profile. Existing approaches are somewhat generic, focusing less on the construction industry than other industrial sectors. This paper probes into CO_2_ generation from the direct activities of China’s construction sector, and CO_2_ emission sources in the six regions, and raises suggestions for integrating key industry policies into China’s quest for environmental sustainability.

## 3. Methodology

### 3.1. Scope of Quantification and Datasets

**Figure 1:**
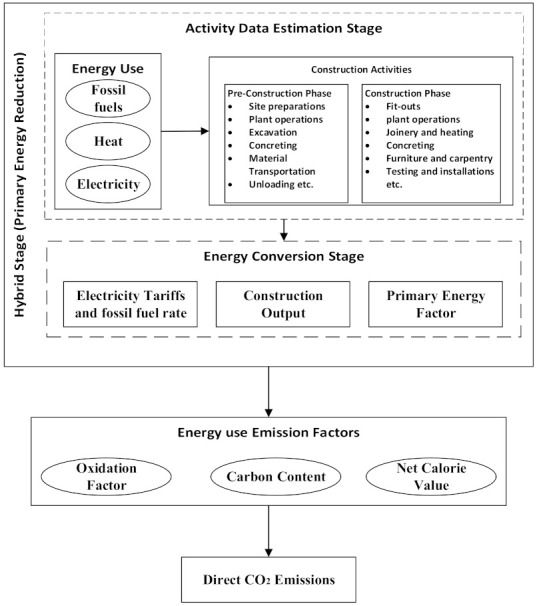
Hybrid quantification of CO_2_ emission in China’s construction sector process map.

Emissions quantification in the construction industry is categorized into direct and indirect emissions [24]. Direct emissions originate from the construction site’s activities, including plant operations, fit-out, excavation, mixing, and concrete placing. In addition to site operation-related emissions, emissions originating from fossil fuels used on the construction site were categorized as direct emissions in this study. Emissions from indirect sources are regarded as those emanating from the downstream sectors of the economy in which materials and other economic inputs in the construction industry are produced and distributed. The study used energy (fossil fuel, electricity, and heat) consumption data for construction-related activities aggregated from the Chinese Energy Yearbook of the National Bureau of Statistics (NBS) as primary data. The data comprise the volume of different fuels consumed on construction sites within China at both national and regional levels, the tariff charged per unit of each fossil fuel consumed, and the emission factors of each fossil fuel. The study adopted the well-established approach of the IPCC emissions quantification, where oxidation factors, carbon content, and net calorie values of each fossil fuel are used in generating the corresponding emission factor. We combined the methodology with other economic and tariff factors to quantify the direct CO_2_ emissions in the sector. Combining the two methods helps cater for double counting problems in using both primary and secondary energy sources. Figure 1 illustrates the process map, explaining the approaches, inputs, and steps employed for the study’s hybrid quantification of direct CO_2_ emissions.

### 3.2. Emission Factors

In GHG emissions calculation, the consumed volume of fuel serves as the activity data in the quantifications. The fuel mix reduces the activity data to suit the model developed, while emission factors are significant to extract the carbon content and oxygenation efficiency of each fuel type used. Emission factors are estimated as the product of the net carbon content (expressed in tonnes of carbon per joule), the net heating value (joules per tonne fuel), the total carbon content (tonnes carbon per tonne fuel), and oxidation rate (carbon oxidized per carbon content). The activity data used in the quantification include fuel consumption data (electricity and heat consumption inclusive) by China’s construction sector in direct site operations, the emission factors for each fuel type, the primary energy factors, and the sector’s total yearly output.

In the Chinese construction sector, there were 26 fuel types consumed between 1997 and 2015. However, due to some fuels’ small quantity consumption and the methodology used in Shan et al. [33], we merged some fuels to have 19 fossil fuels. Notably, the 19 fuels are primary energy sources, including coal, crude oil, and natural gas, while the other fuels are secondary fuels. Shan et al. [33] identified different energy agencies worldwide that publish fossil fuel emission factors for GHGs. Therefore, the emission factors for 17 of the fuels used in this study were retrieved from different sources. Amongst the various sources, IEA and IPCC inventory databases are two of the most renowned emission factor sources for GHGs. However, many countries have revised the original factors domiciled with the IEA and IPCC databases to create country-specific emission factors over the years. The study identified six sources of emission factors, with some having different versions. For heat and electricity CO_2_ emission factors specific to China, we retrieved data from the Climate Transparency [34] report. Table 2 presents the emission factors, the sources, and their retrieved versions. The CO_2_ emissions in the Chinese construction industry were calculated on a national level using these sets of emission factors. However, to uniquely aggregate the direct CO_2_ emissions in each sub-regional classification of the study, the UN-average emission factor was selected as described by Liu et al. [35] to better represent Chinese improved emission technology.

### 3.3. Direct CO_2_ Emissions Calculation

The total direct CO_2_ emissions include the sum of the CO_2_ emission contributions of each fossil fuel used in China’s onsite construction activities. These direct emissions are calculated using the primary energy data collected from the Chinese Energy Yearbook provided by the National Statistics Office. The data comprise each Chinese province’s industrial output, aggregated to regional division. The output data represent both building and civil engineering work carried out in the construction industry within the space of one year throughout the series. Energy tariffs were retrieved from the Census and Economic Information Center (CEIC) global database, showing the average energy rates over an average observation of over 170–198 times from 2003 and 2019 [21]. Equation (1) is the mathematical model used in the calculation of direct CO_2_ emissions.

The model adopted the method used in Acquaye and Duffy [24] to quantify the GHG emissions in the Irish construction industry. Some other studies [36,37] used the IPCC guidelines for quantifying direct CO_2_ emissions without considering specific technological peculiarities in China. We preferred the approach by Acquaye and Duffy [24] because of its comprehensibility, as it is an improvement on the standard direct CO_2_ emissions by the IPCC guidelines. The methodology considers many economic factors such as energy tariffs, delivered energy on construction sites, and energy consumption aggregated directly on construction sites. An advantage of this approach is that it eliminates total reliance on the activity data of energy consumption with no consideration and adjustments for double counting. However, the methodology’s reliance on quantifying CO_2_ emissions using economic factors could be problematic in situations where such data are not published regularly. Additionally, the integrity of published economic data could skew results with reduced chances for validation. Notwithstanding, the methodology presents a robust and comprehensive approach for quantifying direct CO_2_ emissions, considering other economic and environmental construction industry factors. With the improved method, direct integration could be made in overall direct and indirect CO_2_ emissions quantification in the industry, with better data reliability.

We used primary energy factors to convert the direct energy consumed with the energy tariffs and fuel-specific emission factors. The primary energy factor is the ratio of primary energy supplied to the delivered energy. The primary energy factors employed in the study are presented in Table 3.

(1) $$E_{d}=\left[\frac{Q_{\left(e,i\right)}\times T_{\left(e,i\right)}\times P_{\left(e,i\right)}}{O}\right]\times \overset{CO_{2}}{\underset{k}{\sum }}F_{\left(e,i\right)}$$

where *E_d_*—direct CO_2_ emissions (MtCO_2_); *Q*—quantity of energy (fossil fuel and electricity) consumed; *T*—average energy tariff measured in RMB per unit of energy consumed; *P*—primary energy factor; *F*—CO_2_ emission factors estimated in tCO_2_/GJ for electricity and fossil fuels and tCO_2_/m^6^ for gases; e—electricity and heat; *i*—other energy consumed (fossil fuels); and *O*—total construction sector output measured in monetary values (RMB).

### 3.4. Data Management

The various data used in this study, including the construction output data, regional demography, and energy consumption statistics covering 1997 to 2015, were retrieved from the National Statistics Office of China [38] and the CEIC database. The construction industry’s output data are measured over the sector’s GDP contribution to the national economy. The unit of measurement of the economic data is million RMB, with 1997 taken as the constant price to accommodate for inflation’s impact over the investigated period. This study’s energy data include primary and secondary energy sources compiled in China’s NBS and the various regional energy statistics yearbooks. The study’s energy sources include aggregated energy consumption on the construction industry’s sites by fuel type, with renewable energy sources not included. The energy consumption data were made available in million tonnes unit of standard coal equivalent (Mtce) and the gases in million cubic meters unit in calorific value. The secondary data in this paper are consumption data concerning the cement and steel products consumed in the construction industry. The choice of the two datasets was due to the embodied emission potentials of the two construction materials. The two materials could have a combined 80% contribution to CO_2_ emissions in the industry [39,40].

### 3.5. Econometrics Analysis of Regional Direct CO_2_ Emissions in China’s Construction Sector

To further the analysis, we adopted a regression analysis technique to determine the relationship between the direct CO_2_ emissions and the econometrics of the construction industry in China. The extant literature shows that construction materials are significant sources of CO_2_ emissions in the sector [39,41]. We used secondary data of two selected construction materials’ consumption and regional output data of the industry for the relationship testing. We retrieved the yearly construction material consumption and annual regional construction output from the NBS database [38].

**Figure 2:**
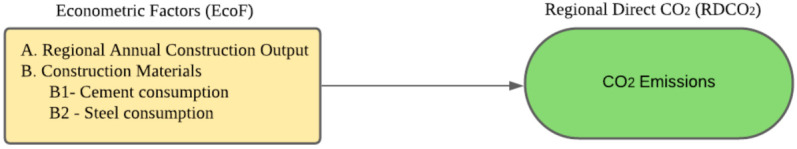
Conceptual EcoF–RDCO_2_ econometric direct CO_2_ emission model in China’s construction sector.

A conceptual model in Figure 2 indicates the interactions among the total direct CO_2_ emissions generated by regional construction industries in China and the three selected econometrics factors. We hypothesized the two groups of factors to affect the regional direct CO_2_ emissions in the industry. The hypotheses are stated below:

**Hypothesis** **1** (**H1**)**:** *Direct CO_2_ emissions in regional construction sectors are significantly related to regional annual construction output.*

**Hypothesis** **2** (**H2**)**:** *Direct CO_2_ emissions in regional construction sectors are significantly related to regional annual cement consumption.*

**Hypothesis** **3** (**H3**)**:** *Direct CO_2_ emissions in regional construction sectors are significantly related to regional annual steel consumption.*

Equations (2) and (3) present the predictive relationship of regional annual construction output, cement, and annual steel consumption (A, B1, and B2) on the dependent variable RDCO_2_ (regional direct CO_2_ emissions) using linear multiple regression analysis.

(2) y=c+mx

(3) $$y=c+m_{1}x_{1}+m_{2}x_{2}+m_{3}x_{3}$$

where *y*—dependent variable, *c*—constant, *m*—slope, and *x*—independent variable.

Equation (3) could be modified to depict the conceptual model interpretation as in Equation (4).

(4) $$RDCO_{2}=c+\beta_{1}A+\beta_{2}B1+\beta_{3}B2$$

where *β*—the beta value of the regression analysis for each independent variable.

## 4. Results

### 4.1. National Direct Energy Consumption

Table 4 shows the distribution of annual energy consumption from direct onsite construction activities in the Chinese construction sector between 1997 and 2015. The data show an increase in the industry’s dependence on coal energy sources between 1997 and 2003, with a significant decrease in 2004. Although there was a steady increase in coal use in the industry from 2004 and 2015, a downward dependence on coal energy sources is observable when the data are juxtaposed with the sector’s output in those years. In Table 4, coke products were shown to contribute less as the years went by, from 126 thousand tonnes in 1997 to 67 thousand tonnes in 2015.

During the investigation period, gasoline consumption as a source of energy on construction sites jumped from 1.1 million tonnes in 1997 to 4.7 million tonnes in 2015. Additionally, there was a significant increase in diesel oil usage, from 1.461 million tonnes in 1997 to 10.35 million tonnes in 2015. There was an increase in other petroleum products between 1997 and 2009, while a downward trend was observed in subsequent years. The energy generated on construction sites using natural gas sources significantly increased between 1997 and 2015, from less than 10 million cubic meters to 620 million cubic meters. The energy input from heat and electricity sources increased by over 360% and 820%, respectively, between 1997 and 2015.

### 4.2. National Direct CO_2_ Emissions in China’s Construction Industry

**Figure 3:**
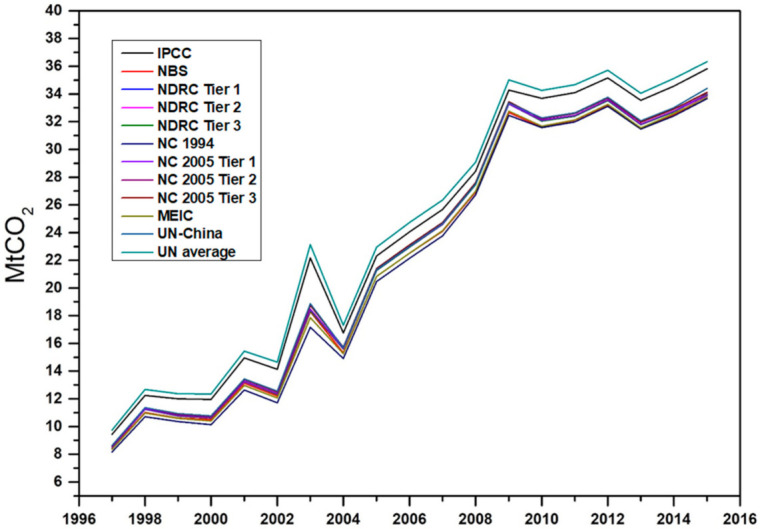
National direct CO_2_ emissions in China’s construction industry.

Emission factors from six official data sources formed the basics of the quantifications in Figure 3. We computed the standard deviation between the CO_2_ emission values, with results ranging between 0.455 and 1.751. Although this range could be taken to be negligible, the size of the dataset involved makes it significant. The IPCC data resulted in the highest emission values compared with the other five sources. The NBS data had the second-highest results, while the National Communication on Climate Change (NC) data factors disaggregated by fuel type and sector had the most negligible CO_2_ emissions results.

According to Liu et al. [35], the UN China data source reflects China’s improved technology. The UN China emission factors were computed to be China-specific and aggregated by Chinese researchers and research agencies. Thus, we chose the UN China CO_2_ emission factors for further analysis in this study. Table 5 shows the direct CO_2_ emissions in the Chinese construction industry using UN China-specific factors. The construction industry in 1997 generated 8.7 million tonnes of CO_2_ direct emissions, with almost 30% from coal sources. The industry’s dependence on coal energy sources continued from 1997 to 2003, where CO_2_ emissions peaked with approximately 58% of national emissions in the sector. A steady reduction in the industry’s reliance on coal energy sources was experienced between 2004 and 2015, reducing coal-generated CO_2_ emissions from 25% to 13% contribution. However, with the decrease in coal reliance, the industry shifted to using more diesel, gasoline, heat, and electricity sources for energy generation. The CO_2_ emissions from these sources increased from 2005 to 2015. Table 5 shows a steady increasing trend of direct CO_2_ emissions from 1997 to 2004, with a massive leap from 2005 up to 2015.

### 4.3. Regional Direct CO_2_ Emissions in China’s Construction Industry

Table 6 shows the aggregation of the direct CO_2_ emissions in the regional construction industries of China. As of 1997, North China’s construction industry had the highest CO_2_ emissions of all regional construction industries, with 3.0 million tonnes of carbon (MtCO_2_). The total direct CO_2_ emissions from North China increased from 3.0 MtCO_2_ to 11 MtCO_2_ in 2010, where the industry experienced its peak emissions. Apart from the surge in CO_2_ emissions in the Northeast construction industry in 2003, the region’s CO_2_ emissions have relatively been kept at a steady rate. The most significant increase in CO_2_ emissions was experienced in East China’s construction industry between 2005 and 2009, before decreasing from 2010 to 2015. The same scenario can be seen to have played out in Southwest and Northwest China’s construction industries, from 1997 to 2015. However, the South Central construction industry results show an increasing trend in CO_2_ emissions instead of other regional construction industries.

**Figure 4:**
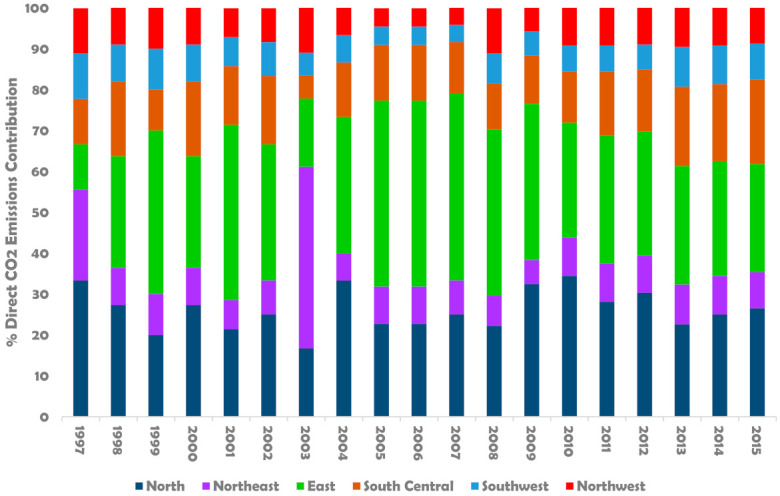
Changes in regional construction industry contributions to national direct CO_2_ emissions from 1997 to 2015.

Figure 4 illustrates the regional construction industries’ percentage contributions to China’s construction sector CO_2_ emissions from 1997 to 2015. Although a plunge in CO_2_ emissions from the East resulted in a corresponding surge in the North in 2003, the East’s construction industry contributed more than other regions from 1997 to 2015. As of 1997, the construction industry in the East, South Central, Southwest, and Northwest had approximately equal contributions to the industry’s direct CO_2_ emissions stock. However, the direct CO_2_ emission generated in the East’s construction industry has been more than in the Southwest and South Central regions in recent years.

**Figure 5:**
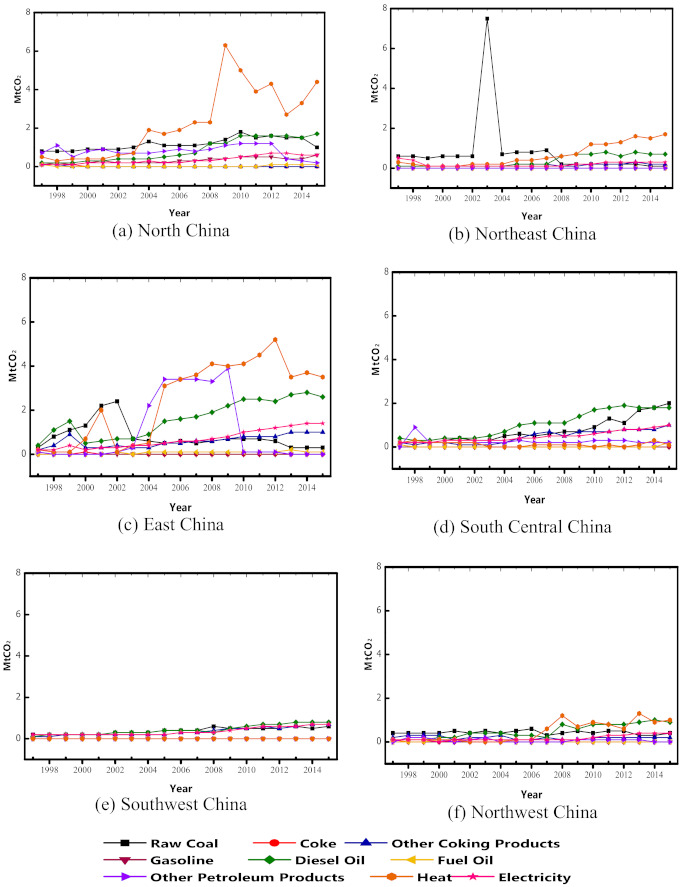
Energy sources of direct CO_2_ emissions in the regional construction industries of China.

Parallel to Figure 4, Figure 5a–f illustrates the energy sources for direct CO_2_ emissions from the regional construction industries in China. The share of each fuel considered in this study is presented as total direct CO_2_ emissions in the regional construction industries. In the North construction industry’s direct CO_2_ emissions inventory, there is a spike in the emissions originating from the heat sources used on construction sites. Similarly, heat-related CO_2_ emissions increase in East and Northeast China’s construction industries, while its proportion in other regions is relatively stable. In all regions, the relatively low emissions are generated from natural gases because of their relatively low carbon content. However, the CO_2_ emissions resulting from diesel oil and other petroleum products increased in the emission inventories. Appreciably, the direct CO_2_ emissions from raw coal are on a massive decline in all regions, except for a recent increase in the South Central construction industry observable from 2010. In the Northeast region, Figure 5b indicates a spike in coal-based CO_2_ emissions in 2003. However, in 2004, there was an observable drop in coal-related CO_2_ emissions and a continuous drop in subsequent years, as seen in other regions. The decrease in raw coal contribution to direct CO_2_ emissions in the construction industry connotes the shift from absolute dependence on coal as the primary energy generation source in the Chinese economy.

### 4.4. Econometric Analysis of Regional Direct CO_2_ Emissions in China’s Construction Sector

We conducted a Pearson correlation analysis to establish the relationship between the dependent and the independent variables. The study identified the direction and strength of the relationship between the variables, with the significance reported at the 0.01 level (99% confidence interval). Presented in Table 7 are the correlation analysis reports in each of the six regional construction sectors in China. Significant relationships were found between the variables in all the regions, except in the Northeast regional construction sector, which had no factors correlated with the others. The results in Table 7 show that the relationship between the factors is strong, above the medium effect size of 0.3 recommended in Leung et al. [42]. Therefore, we used multiple linear regression analysis to determine the effects of the factors on CO_2_ emissions in the regions.

**Figure 6:**
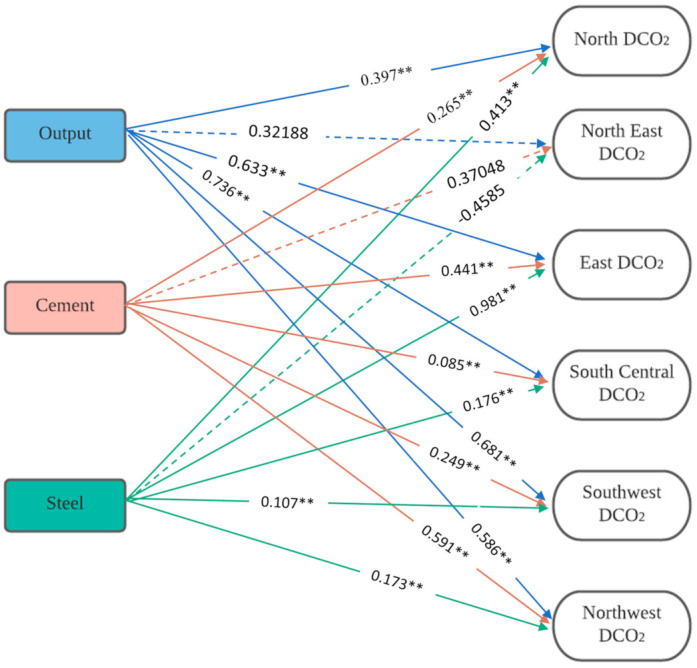
EcoF–RDCO2 direct CO_2_ emission model in China’s construction sector. ** Correlation is significant at the 0.01 level (2-tailed).

Regression, the statistical tool used, explains each independent variable’s unique contribution and the amount of variance their combination can predict [42,43]. We inputted ‘EcoF’ factors for each region as independent variables, and RDCO_2_ were the dependent variables. Table 8 presents the resulting models. The variance explained by the models ranges from 86% to 98%, indicating the strength of the models. We discontinued modeling for northeast China because of its lack of regression significance, confirming the correlation analysis. Expressed in Equations (5)–(9) are direct CO_2_ emissions models for each of the five regional construction sectors extracted from the regression model in Table 8. The models depict the positive predictive powers of the EcoF factors in the five regions, indicating an increase in direct CO_2_ emissions if any of the three factors increases and vice versa. Figure 6 shows a pictorial illustration of the relationship and the predictive correlational values between the dependent and the independent variables in each region of China’s construction sector. The relationship between the independent variables in the Northeast region is drawn in broken lines to depict the insignificance and lack of predictive appropriateness with the direct CO_2_ emissions in the region.

(5) $$North=1.959882+0.396789\;\left(A\right)+0.265402\;\left(B1\right)+0.413016\;\left(B2\right)$$

(6) $$East=2.714384+0.63278\;\left(A\right)+0.441105\;\left(B1\right)+0.980634\;\left(B2\right)$$

(7) $$South\;Central=1.225556+0.739552\;\left(A\right)+0.08464\;\left(B1\right)+0.176465\;\left(B2\right)$$

(8) $$Southwest=0.609027+0.681099\;\left(A\right)+0.249427\;\left(B1\right)+0.10714\;\left(B2\right)$$

(9) $$Northwest=0.814057+0.586157\;\left(A\right)+0.591359\;\left(B1\right)+0.1734\;\left(B2\right)$$

## 5. Discussion of Findings

The Chinese construction industry’s growth was observed in many other studies [44,45]. The Chinese government’s resolve to re-strategize the sector towards a sustainable environment was a focal point of this study. The results show an increase in the size of the construction industry in the six regions. The direct CO_2_ emissions from the construction sector in the six regions reflect such growth in output. The results show an increase in the direct CO_2_ emissions produced in the years considered, the largest being observed in the Northeast China construction industry in 2003. The construction industry’s CO_2_ emissions increased significantly around 2001, with all six regional construction sectors contributing more to the national emissions inventories. This supports the view from previous studies suggesting China’s joining of the WTO was instrumental in China’s CO_2_ emissions growth [46,47,48]. The admittance of China into the WTO significantly increased export in the construction industry, thereby increasing energy consumption. The resultant increase in energy consumption and export in the sector was observed in a corresponding rise in coal consumption between 2000 and 2003. The construction sector in North China contributes an average of 30% of the CO_2_ emissions in the industry. The lowest emissions are observed in the Southwest construction sector. In 2003, CO_2_ emissions in China experienced a spike in the Northeast region in response to a peak in coal production in China, with most being locally consumed [49]. Although the sharp increase in coal-related CO_2_ emissions in the Northeast construction industry was adequately regulated in 2004, the study observed a stable rise in other regional construction industries, especially in the South Central provinces.

Nonetheless, the results show a gradual move away of the construction industry in the six regions from primary dependence on coal as the primary energy source on construction sites. On the national scale, coal’s influence as the primary fuel source for plants and energy sources has reduced over the years. Although the South Central and Southwestern construction industries are yet to significantly cut their dependence on coal and fossil fuels as much as other regions, the overall improvements in the construction industry’s energy profile are targeted at reducing CO_2_ emissions from the sector. The study also shows that the most significant source of direct CO_2_ emissions is heat and electricity use on construction sites. Electricity and heat are the two most potent energy sources of the construction industry, amounting to over 30% of the total CO_2_ emissions generated in the industry during the investigation period. The result resonates with Fe et al.’s [50] claims that China is shifting away from the usual energy generation means such as coal, crude oil, and other fossil fuels to electricity and more secondary energy sources. The electricity-related CO_2_ emissions profile in the North indicates a move away from coal between 2007 and 2009. Heat sources are the most significant sources of CO_2_ emissions in four of the six regional construction industries.

Our results show an improved and more efficient construction industry in China instead of indications from past studies, in which the sector is regarded as one with very low carbon efficiency [51,52]. Wang et al. [52] categorized the Chinese construction sector as a low-carbon industry with high potential to increase national CO_2_ emissions stock due to massive construction activities and a low technological adoption to improve carbon efficiency. Meanwhile, the rise in direct CO_2_ emissions may not be directly linked to the industry’s technology. Still, the overall increase in construction output has resulted in more fuel sources in the sector’s production processes at different stages and in various regions of China. The characteristics of the industry’s dependence on coal and crude oil for construction sites activities pose a danger to carbon reduction strategies [53]. In recent years, Chen et al. [54] regarded the construction sector as the pillar of all Chinese national economic development. With such growth in the sector, it is an imminent priority to focus more on reducing energy consumption and non-renewable energy sources with improved carbon-efficient technologies.

The analysis results show significant relationships between construction material (cement and steel products) consumption, annual construction output, and direct CO_2_ emissions in the regional construction sectors in China. The results align with past studies on the effects of construction materials on CO_2_ emissions in China and other countries. De Wolf et al. [55] averred the importance of materials such as cement and steel in driving increased embodied CO_2_ emissions. In the same vein, Galvez-Martos et al. [56] argued that the chemical composition of cement is a critical driver of CO_2_ emissions in the construction sector. Additionally, many studies campaigned for process change in the industry to reduce cement and steel usage to manage CO_2_ emissions effectively [57,58,59]. Inductively, based on the regression results from the study, an increase in cement and steel usages can increase direct CO_2_ emissions in the industry.

However, as China continues to expand in construction and infrastructural developments, more cement and steel products will be needed to satisfy the increased demand in construction product delivery [34]. Cement and steel are two of the most critical construction materials required in the construction process [60], indicating an expected rise in consumption due to the increasing urbanization and economic development boom in China. Therefore, it is expedient to find methods of improving the carbon-content- and CO_2_-emission-generating potentials of both materials by finding alternative construction materials that can serve similar functional requirements and enhance manufacturing. Achieving acceptable global sustainability practices in the construction sector of China depends on the efficiency of the industry in managing its CO_2_ emissions profile, with more focus on construction material improvements.

## 6. Policy Implications

### 6.1. Increased Focus on the Construction Sector

The Chinese construction industry has received underwhelming attention in emissions inventory studies and policy drive. Many government policies focus on heavy industries such as manufacturing, oil, and gas, and other sectors believed to have higher carbon intensities. However, the construction industry has significant input in the country’s emissions. In quantifying CO_2_ emissions originating from the Chinese construction sector, many researchers have focused on the downstream sectors of the Chinese economy, with little detail on the direct CO_2_ emissions from construction activities. To fully understand the nature of emissions in the construction industry in China, emphasis needs to be placed on regular quantifications of emissions generated directly on construction sites in China. We recommend construction-industry-focused policies to guide the emissions emanating in construction processes in China as obtainable in other parts of the world. While emission reductions are focused on the construction industry, it is, however, critical that geospatial considerations are made in GHG policy formulations for the construction industry to help mitigate climate change. Our study presents a comprehensive yet straightforward approach to generating preliminary direct CO_2_ emissions in the construction sector for fast checking of and indications on the needed carbon efficiency improvement strategies.

### 6.2. Redistribution of Uneven Energy Sources in Chinese Regions

Energy resource generation and distribution in the industry need to be extensively studied. The alarming reliance of some regional construction industries on coal and fossil fuels is because of the high concentration of such fuel types in the region, especially in North and Northeast China, with significant coal and oil deposits. The uneven distribution of energy sources in the areas threatens to disrupt sustainability goals in the regions. On a national level, China needs mega-projects to redistribute energy resources between the regions to forestall absolute dependence on the most abundant energy resources in the regions, solving the uneven energy distribution problem.

### 6.3. Construction Materials Improvement and Increased Renewable Energy Use

Globally, the concept of emission reduction, technology innovations, energy conservation, and renewable energy dependence has been proposed in construction sectors. The construction industry in China needs to focus more on material-improving technologies, as most emissions in the sector are material-originated. Cement and steel consumption composition need to be technologically enhanced to reduce their carbon contents. There is a need for continuous process change to shift from fossil-fuel- and electricity-driven machinery to renewable energy use. Increased renewable energy use would alleviate dependence on primary energy sources, reducing the industry’s environmental risks.

## 7. Conclusions

The study identified the North construction sector as the most critical emitter of CO_2_. The study shows a gradual shift of China’s construction industry from coal and crude oil as the primary sources of energy generation on construction sites, and the corresponding reduction in CO_2_ emissions from both sources on a national level. However, two construction sector regions still depended more on coal and crude oil as direct energy sources. The study reflects that China’s commitment to the global climate change campaign lacks sufficient policies specific to the construction sector. To achieve sustainability, all industrial sectors (construction industry inclusive) require specific policies to manage CO_2_ emission profiles and energy resource redistribution amongst the regions. The study establishes the relationships between direct CO_2_ emissions, annual construction output, and construction materials. Cement and steel consumption directly affect the direct CO_2_ emissions generated on construction sites in China’s five regional construction industries. Considering the population of China, the increase in emissions in China can be attributed to an increased urbanization rate and a corresponding rise in building stock demand in the regions. Although the study showed a positive relationship between construction output due to increased urbanization and direct CO_2_ emissions, it will be prejudicial to measure the emission profile of China solely on the amount of generated CO_2_ emissions from the country without considering other economic indices.

The study assessed direct CO_2_ emissions based on three selected factors; however, to generate a perfect predictive model, China’s government and industrial sectors need to consider other economic indices and environmental sustainability campaigns. As a recommendation, studies on carbon emissions quantification in China need to adopt specific population density indices in future research to adequately represent China’s emission profile. The study quantified direct CO_2_ emissions from the Chinese construction industry, which amounted to 10–15% of the sector’s total CO_2_ emissions. Consequently, future work should extend the methodology to quantify indirect CO_2_ emission spills from other Chinese economic sectors. The study faced a limitation of data availability during the investigation period. The last year of the investigation was 2017 due to data unavailability and consistency. There is a need to harmonize Chinese data and create a data repository to enhance the data available for research purposes.

## Figures and Tables

**Table 1:** Provinces/regions/municipal demography of China.

Region	Area	Provinces
North China	1,556,061 km^2^	Beijing
		Tianjin
		Hebei
		Shanxi
		Inner Mongolia
Northeast China	793,300 km^2^	Liaoning
		Jilin
		Heilongjiang
East China	795,837 km^2^	Shanghai
		Jiangsu
		Zhejiang
		Anhui
		Fujian
		Jiangxi
		Shandong
South Central China	1,014,354 km^2^	Henan
		Hubei
		Hunan
		Guangdong
		Guangxi
		Hainan
Southwest China	2,365,900 km^2^	Chongqing
		Sichuan
		Guizhou
		Yunnan
Northwest China	3,107,701 km^2^	Shaanxi
		Gansu
		Qinghai
		Ningxia
		Xinjiang

**Table 2:** Emission factors for fuels specific to China’s construction industry.

	Sector-Fuel	Unit	IPCC	NBS	NDRC Tier 1	NDRC Tier 2	NDRC Tier 3	NC 1994	NC 2005 Tier 1	NC 2005 Tier 2	NC 2005 Tier 3	MEIC	UN China	UN Average
1	Raw Coal	tCO_2_/GJ	0.713009	0.518263	0.518263	0.512957	0.512957	0.456565	0.519570	0.532320	0.532320	0.491710	0.539426	0.755940
2	Cleaned Coal	tCO_2_/GJ	0.701249	0.656013	0.577971	0.591437	0.577971	0.580206	0.581132	0.589772	0.592755	0.679613	0.560334	0.785240
3	Other Washed Coal	tCO_2_/GJ	0.701249	0.383313	0.577971	0.597797	0.577971	0.452007	0.581132	0.589772	0.592755	0.397103	0.560334	0.785240
4	Briquettes	tCO_2_/GJ	0.652327	0.537510	0.527362	0.551457	0.527362	0.435562	0.527362	0.527990	0.527362	0.459095	0.539426	0.755940
5	Coke	tCO_2_/GJ	0.806971	0.777999	0.777999	0.754949	0.777999	0.814669	0.738059	0.776062	0.761021	0.725802	0.770203	0.770203
6	Coke Oven Gas	tCO_2_/m^6^	2.252052	2.192480	2.333106	2.191706	2.333106	3.228984	2.405264	2.405264	2.405264	2.536039	2.274800	2.274800
7	Other Gas	tCO_2_/m^6^	2.252052	1.018055	1.903300	1.884074	1.903300	1.009710	1.903300	1.903300	1.903300	0.792531	2.274800	2.274800
8	Other Coking Products	tCO_2_/GJ	1.098306	0.780114	0.780114	0.822056	0.780114	0.711614	0.528891	0.776062	0.761021	0.832443	1.109400	1.109400
9	Crude Oil	tCO_2_/GJ	0.837540	0.822872	0.838693	0.796696	0.838693	0.820613	0.838693	0.837838	0.837838	0.832443	0.846000	0.846000
10	Gasoline	tCO_2_/GJ	0.831330	0.798743	0.829786	0.829786	0.829786	0.829786	0.829786	0.829786	0.829786	0.857407	0.848591	0.848591
11	Kerosene	tCO_2_/GJ	0.845559	0.828326	0.859558	0.859558	0.859558	0.859558	0.859558	0.859558	0.859558	0.857407	0.842388	0.842388
12	Diesel Oil	tCO_2_/GJ	0.859914	0.844339	0.857761	0.857761	0.857761	0.886861	0.857761	0.858235	0.859511	0.849085	0.858419	0.858419
13	Fuel Oil	tCO_2_/GJ	0.843916	0.864671	0.831049	0.831049	0.831049	0.831049	0.831049	0.834893	0.835289	0.832443	0.852440	0.852440
14	LPG	tCO_2_/GJ	0.805424	0.854448	0.805595	0.797457	0.805595	0.805595	0.805595	0.804781	0.804781	0.998927	0.783288	0.783288
15	Refinery Gas	tCO_2_/m^6^	0.769379	0.829819	0.829729	0.821348	0.829729	0.624673	0.829729	0.828891	0.828891	0.916830	0.657453	0.657453
16	Other Petroleum Products	tCO_2_/GJ	0.792000	0.819594	0.883960	0.883960	0.883960	0.787724	0.883960	0.883960	0.883960	0.832443	0.849920	0.849920
17	Natural Gas	tCO_2_/GJ	0.521057	0.590459	0.590459	0.583731	0.590459	0.590733	0.590459	0.590459	0.590459	0.590282	0.526320	0.526320
21	Electricity	tCO_2_/GJ	0.623600	0.623600	0.623600	0.623600	0.623600	0.623600	0.623600	0.623600	0.623600	0.623600	0.623600	0.623600

**Table 3:** Primary energy factors (Acquaye and Duffy, 2010, Climate Transparency, 2018).

Countries	Mains Gas	LPG	Oil—General	Diesel or Heating Oil	Fuel Oil	Coal—General	Biomass—General	Wood—General	Wood Pellets	Grid Electricity	Heating—General
EU countries in average	1.00–1.26	1.00–1.20	1.00–1.23	1.00–1.14	1.00–1.20	1.00–1.46	0.01–1.10	0.01–1.20	0.01–1.26	1.5–3.45	0.15–1.50
CEN (nonrenewable) defaults	1.1	1.1	1.1	1.1	1.1	1.1	0.2	0.2	0.2	2.3	1.3
China	1.1	1.1	1.1	1.1	1.1	1.1	0.2	0.2	0.2	2.93	1.3

**Table 4:** National construction industry’s energy and fossil fuel consumption (Shan et al., 2018).

Year	Raw Coal	Cleaned Coal	Other Washed Coal	Briquettes	Coke	Coke Oven Gas	Other Gas	Other Coking Products	Crude Oil	Gasoline	Kerosene	Diesel Oil	Fuel Oil	LPG	Refinery Gas	Other Petroleum Products	Natural Gas	Heat	Electricity
	10^4^ tn	10^4^ tn	10^4^ tn	10^4^ tn	10^4^ tn	10^8^ cu.m	10^8^ cu.m	10^4^ tn	10^4^ tn	10^4^ tn	10^4^ tn	10^4^ tn	10^4^ tn	10^4^ tn	10^4^ tn	10^4^ tn	10^8^ cu.m	10^10^ kj	10^4^ kwh
1997	497.0	0.7	8.5	0.0	12.6	0.0	0.0	5.5	3.1	111.6	4.2	146.1	19.2	5.1	0.0	89.4	0.0	207.3	197.60
1998	601.3	0.1	10.6	0.0	14.6	0.0	0.0	6.3	2.2	134.0	3.4	240.9	16.1	5.7	0.0	256.6	0.1	166.1	195.6
1999	651.2	4.3	11.7	0.0	16.4	0.0	0.0	0.0	3.2	199.3	3.3	293.4	16.4	7.8	0.0	98.1	0.7	132.7	171.60
2000	716.0	4.0	3.5	0.0	22.7	0.0	0.0	0.0	7.6	137.7	2.1	197.7	24.1	9.0	0.0	121.8	0.8	232.7	158.0
2001	916.0	4.0	4.7	0.0	23.6	0.0	0.0	0.0	11.3	129.7	3.5	213.8	24.2	7.5	0.0	128.4	0.5	455.0	162.2
2002	957.7	3.3	3.6	0.1	23.1	0.0	0.0	0.0	14.8	144.2	2.8	251.8	28.3	13.1	0.0	129.3	0.5	162.2	188.4
2003	1963.1	3.0	4.1	5.2	20.8	0.0	0.0	0.8	2.0	146.1	0.9	276.6	26.3	7.9	0.0	166.0	1.4	216.0	215.0
2004	737.8	3.1	4.5	0.3	16.4	0.0	0.0	0.0	0.0	155.0	0.4	325.3	26.7	8.8	0.0	360.5	1.4	418.6	232.5
2005	737.7	3.4	5.7	3.8	28.5	0.0	0.0	0.6	0.0	207.7	0.6	444.5	14.2	6.5	0.0	522.0	1.4	860.9	238.4
2006	776.4	3.9	5.4	0.9	27.3	0.0	0.0	0.6	0.0	232.7	0.8	497.5	16.7	7.6	0.0	526.2	1.6	951.1	271.9
2007	746.3	0.1	30.4	2.6	27.7	0.0	0.0	1.1	0.0	276.6	0.3	502.7	15.8	7.2	0.0	520.4	3.6	1118.9	312.2
2008	692.6	1.2	29.4	3.6	18.1	0.0	0.0	1.2	0.0	247.5	7.1	703.3	26.2	4.7	0.0	525.1	4.3	1329.8	350.8
2009	729.4	1.1	32.0	5.6	5.7	0.0	0.1	13.5	0.0	301.2	7.8	791.3	23.6	6.0	0.0	607.8	4.1	1874.2	396.7
2010	845.1	1.4	67.0	5.5	5.8	0.1	0.0	0.7	0.0	350.6	6.6	939.0	21.1	5.8	0.0	189.2	6.6	1788.2	501.9
2011	904.9	5.5	0.5	14.9	4.9	0.2	0.1	0.7	0.0	360.3	8.7	984.0	20.8	5.8	0.0	189.3	6.4	1674.0	597.2
2012	868.8	6.0	0.5	22.1	6.3	0.0	0.1	0.7	0.0	363.8	5.7	977.9	18.6	5.3	0.0	193.9	7.3	1842.3	635.0
2013	875.7	6.8	20.5	15.1	7.6	0.0	0.0	0.8	0.0	425.5	8.5	1039.6	30.7	10.9	0.0	72.9	3.1	1468.4	690.0
2014	875.6	63.4	20.0	16.8	9.7	0.0	0.0	0.9	0.0	432.1	7.6	1030.4	24.6	14.7	0.0	62.3	3.0	1534.0	741.1
2015	841.0	8.1	18.8	18.0	6.7	0.0	0.0	46.3	0.0	468.1	10.8	1035.6	44.2	12.4	0.0	43.5	6.2	1712.7	727.8

**Table 5:** National Chinese construction industry CO_2_ emissions based on UN China emission factors (MtCO_2_).

Year	Raw Coal	Cleaned Coal	Other Washed Coal	Briquettes	Coke	Coke Oven Gas	Other Gas	Other Coking Products	Crude Oil	Gasoline	Kerosene	Diesel Oil	Fuel Oil	LPG	Refinery Gas	Other Petroleum Products	Natural Gas	Heat	Electricity	Total
1997	2.6	0.0	0.0	0.0	0.3	0.0	0.0	0.1	0.0	1.0	0.0	1.2	0.2	0.0	0.0	0.8	0.0	1.3	1.2	8.7
1998	3.2	0.0	0.2	0.0	0.1	0.0	0.0	0.1	0.0	1.2	0.0	2.0	0.1	0.0	0.0	2.2	0.0	1.0	1.2	11.3
1999	3.5	0.0	0.1	0.0	0.1	0.0	0.0	0.0	0.0	1.7	0.0	2.5	0.1	0.1	0.0	0.9	0.0	0.8	1.1	10.9
2000	3.8	0.0	0.0	0.0	0.1	0.0	0.0	0.0	0.1	1.2	0.0	1.7	0.2	0.1	0.0	1.1	0.0	1.5	1.0	10.8
2001	4.9	0.0	0.0	0.0	0.0	0.0	0.0	0.0	0.1	1.1	0.0	1.8	0.2	0.1	0.0	1.1	0.0	2.8	1.0	13.1
2002	5.1	0.0	0.0	0.0	0.1	0.0	0.0	0.0	0.1	1.2	0.0	2.2	0.2	0.1	0.0	1.1	0.0	1.0	1.2	12.3
2003	10.6	0.0	0.0	0.0	0.0	0.0	0.0	0.0	0.0	1.2	0.0	2.4	0.2	0.1	0.0	1.4	0.0	1.3	1.3	18.5
2004	3.9	0.0	0.0	0.0	0.0	0.0	0.0	0.0	0.0	1.3	0.0	2.8	0.2	0.1	0.0	3.1	0.0	2.6	1.5	15.5
2005	4.0	0.0	0.0	0.0	0.0	0.0	0.0	0.0	0.0	1.7	0.0	3.8	0.1	0.1	0.0	4.4	0.0	5.4	1.5	21.0
2006	4.2	0.0	0.0	0.0	0.0	0.0	0.0	0.0	0.0	1.9	0.0	4.2	0.1	0.1	0.0	4.4	0.0	5.9	1.7	22.5
2007	4.0	0.0	0.0	0.0	0.0	0.0	0.0	0.0	0.0	2.3	0.0	4.3	0.1	0.1	0.0	4.4	0.0	7.0	1.9	24.1
2008	3.7	0.0	0.0	0.0	0.0	0.0	0.0	0.0	0.0	2.1	0.1	6.1	0.2	0.0	0.0	4.4	0.0	8.3	2.2	27.1
2009	4.0	0.0	0.0	0.0	0.0	0.0	0.0	0.1	0.0	2.5	0.1	6.8	0.2	0.0	0.0	5.2	0.0	11.7	2.5	33.1
2010	4.5	0.0	0.0	0.0	0.0	0.0	0.0	0.0	0.0	2.9	0.1	8.0	0.2	0.0	0.0	1.6	0.0	11.2	3.1	31.6
2011	4.8	0.0	0.0	0.1	0.0	0.0	0.0	0.0	0.0	3.0	0.1	8.4	0.2	0.0	0.0	1.7	0.0	10.4	3.7	32.4
2012	4.6	0.0	0.0	0.1	0.0	0.0	0.0	0.0	0.0	3.0	0.0	8.3	0.2	0.0	0.0	1.7	0.0	11.5	4.0	33.4
2013	4.6	0.0	0.0	0.1	0.0	0.0	0.0	0.0	0.0	3.5	0.1	8.9	0.3	0.1	0.0	0.7	0.0	9.2	4.3	31.8
2014	4.6	0.4	0.0	0.1	0.0	0.0	0.0	0.0	0.0	3.6	0.1	8.9	0.2	0.1	0.0	0.5	0.0	9.6	4.6	32.7
2015	4.6	0.0	0.0	0.1	0.0	0.0	0.0	0.5	0.0	3.9	0.1	8.9	0.4	0.1	0.0	0.4	0.0	10.7	4.5	34.2

**Table 6:** Regional construction industries’ direct CO_2_ emission inventories (MtCO_2_).

Year	North China	Northeast China	East China	South Central China	Southwest China	Northwest China	National Average
1997	2.7	1.6	1.4	1.4	0.6	1.0	8.7
1998	3.1	1.4	2.6	2.0	0.7	1.2	11.0
1999	2.3	0.9	4.0	1.3	0.8	1.2	10.5
2000	2.8	1.0	3.3	1.6	0.8	1.2	10.7
2001	3.0	1.0	5.5	1.6	0.8	1.1	13.0
2002	3.0	1.1	4.4	1.5	1.0	1.3	12.3
2003	3.2	8.0	2.8	1.4	1.0	1.5	17.9
2004	4.8	1.2	5.0	1.9	1.0	1.3	15.2
2005	4.5	1.7	9.6	2.7	1.2	1.1	20.8
2006	5.0	1.7	10.2	2.9	1.4	1.2	22.4
2007	5.5	1.9	10.5	3.2	1.4	1.4	23.9
2008	6.3	1.6	11.3	3.1	1.6	2.6	26.5
2009	10.8	1.9	12.5	3.6	1.9	2.1	32.8
2010	10.6	2.5	9.3	4.2	2.1	2.6	31.3
2011	9.3	2.7	9.8	5.0	2.4	2.7	31.9
2012	10.0	2.6	10.4	5.0	2.3	2.5	32.8
2013	7.4	3.2	9.1	5.5	2.6	3.2	31.0
2014	7.7	2.8	9.4	6.2	2.7	2.8	31.6
2015	8.6	3.0	8.9	6.5	3.3	2.9	33.2

**Table 7:** EcoF–RDCO2 direct CO_2_ emissions in China’s construction sector correlation analysis report.

		Regional Direct CO_2_ Emissions						
Variables	Factors	North	Northeast	East	South Central	Southwest	Northwest	National
RDCO_2_	Direct CO_2_	1	1	1	1	1	1	1
A	Annual Construction Output	0.832 **	0.265044	0.630 **	0.983 **	0.973 **	0.922 **	0.879 **
B1	Cement Consumption	0.608 **	0.070884	0.854 **	0.922 **	0.918 **	0.906 **	0.947 **
B2	Steel Consumption	0.892 **	0.053849	0.838 **	0.961 **	0.858 **	0.912 **	0.979 **

** Correlation is significant at the 0.01 level (2-tailed).

**Table 8:** EcoF–RDCO2 direct CO_2_ emission regression model in China’s construction sector.

									ANOVA	
Model	Factors	Code	Constant (c)	ß	S.E.	R	R^2^	ΔR^2^	F	Sig.
North	Output	A	1.959882	0.3968	1.2073	0.927	0.860018	0.832021	30.71877	0.000
	Cement	B1		0.2654						
	Steel	B2		0.4130						
*Northeast*	*Output*	*A*	*1.808724*	*0.3219*	*1.6544*	*0.295*	*0.086883*	*0.09574*	*0.475752*	*0.7038*
	*Cement*	*B1*		*0.3705*						
	*Steel*	*B2*		*−0.4585*						
East	Output	A	2.714384	0.6328	1.7063	0.894	0.799625	0.75955	19.9532	0.000
	Cement	B1		0.4411						
	Steel	B2		0.9806						
South Central	Output	A	1.225556	0.7396	0.3047	0.987	0.974397	0.969276	190.2864	0.000
	Cement	B1		0.0846						
	Steel	B2		0.1765						
Southwest	Output	A	0.609027	0.6811	0.1126	0.992	0.983579	0.980295	299.496	0.000
	Cement	B1		0.2494						
	Steel	B2		0.1071						
Northwest	Output	A	0.814057	0.5862	0.2406	0.958	0.91824	0.901888	56.15448	0.000
	Cement	B1		0.5914						
	Steel	B2		0.1734						

ß = independent variable coefficients, S.E. = standard error, ΔR^2^ = adjusted R^2^

## Data Availability

Data is contained within the article.
